# Attitude of aspiring orthopaedic surgeons towards artificial intelligence: a multinational cross-sectional survey study

**DOI:** 10.1007/s00402-024-05408-0

**Published:** 2024-08-10

**Authors:** Johannes Pawelczyk, Moritz Kraus, Larissa Eckl, Stefan Nehrer, Matthias Aurich, Kaywan Izadpanah, Sebastian Siebenlist, Marco-Christopher Rupp

**Affiliations:** 1 Klinikum rechts der Isar, Sektion Sportorthopädie, Technische Universität München, Haus 524, Ismaninger Str. 22, 81675 Munich, Germany; 2 Schulthess Klinik, Abteilung für Schulter- und Ellenbogenchirurgie, Zurich, Switzerland; 3 Klinische Abteilung für Orthopädie und Traumatologie, Universitätsklinikum Krems, Krems an der Donau, Austria; 4 Zentrum für Regenerative Medizin, Universität für Weiterbildung Krems, Krems an der Donau, Austria; 5 Fakultät für Gesundheit und Medizin, Universität für Weiterbildung Krems, Krems an der Donau, Austria; 6 Universitätsklinikum Halle (Saale), Halle, Germany; 7 BG Klinikum Bergmannstrost, Halle, Germany; 8 Klinik für Orthopädie und Unfallchirurgie, Universitätsklinikum Freiburg, Medizinische Fakultät, Albert-Ludwigs-Universität Freiburg, Freiburg, Germany

**Keywords:** Artificial intelligence, Machine learning, Digital health, Orthopaedic surgery, Survey

## Abstract

**Introduction:**

The purpose of this study was to evaluate the perspectives of aspiring orthopaedic surgeons on artificial intelligence (AI), analysing how gender, AI knowledge, and technical inclination influence views on AI. Additionally, the extent to which recent AI advancements sway career decisions was assessed.

**Materials and methods:**

A digital survey was distributed to student members of orthopaedic societies across Germany, Switzerland, and Austria. Subgroup analyses explored how gender, AI knowledge, and technical inclination shape attitudes towards AI.

**Results:**

Of 174 total respondents, 86.2% (n = 150) intended to pursue a career in orthopaedic surgery and were included in the analysis. The majority (74.5%) reported ‘basic’ or ‘no’ knowledge about AI. Approximately 29.3% believed AI would significantly impact orthopaedics within 5 years, with another 35.3% projecting 5–10 years. AI was predominantly seen as an assistive tool (77.8%), without significant fear of job displacement. The most valued AI applications were identified as preoperative implant planning (85.3%), administrative tasks (84%), and image analysis (81.3%). Concerns arose regarding skill atrophy due to overreliance (69.3%), liability (68%), and diminished patient interaction (56%). The majority maintained a ‘neutral’ view on AI (53%), though 32.9% were ‘enthusiastic’. A stronger focus on AI in medical education was requested by 81.9%. Most participants (72.8%) felt recent AI advancements did not alter their career decisions towards or away from the orthopaedic specialty. Statistical analysis revealed a significant association between AI literacy (p = 0.015) and technical inclination (p = 0.003). AI literacy did not increase significantly during medical education (p = 0.091).

**Conclusions:**

Future orthopaedic surgeons exhibit a favourable outlook on AI, foreseeing its significant influence in the near future. AI literacy remains relatively low and showed no improvement during medical school. There is notable demand for improved AI-related education. The choice of orthopaedics as a specialty appears to be robust against the sway of recent AI advancements.

**Level of evidence:**

Cross-sectional survey study; level IV.

**Supplementary Information:**

The online version contains supplementary material available at 10.1007/s00402-024-05408-0.

## Introduction

**Fig. 1:**
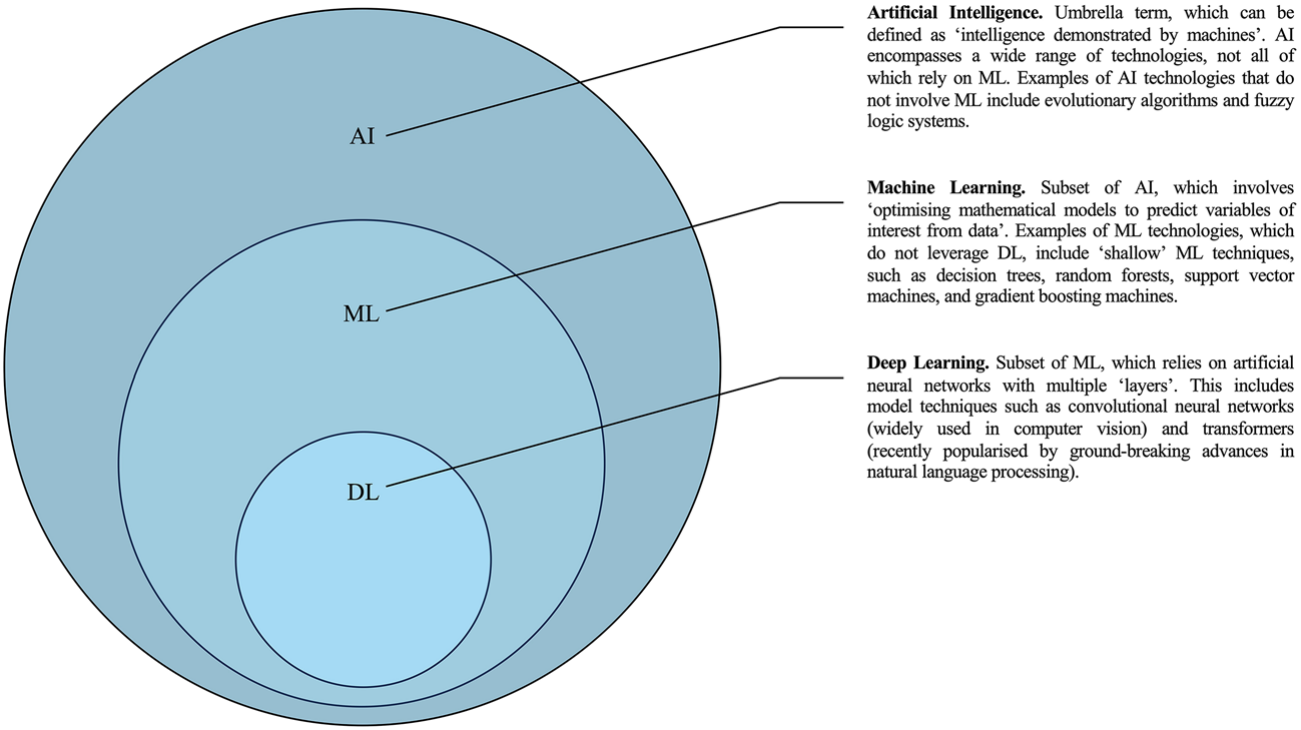
Important terminology in AI. Set diagram describing key nomenclature in AI. *AI* artificial intelligence; *ML* machine learning; *DL* deep learning

The field of artificial intelligence (AI) has made unprecedented progress in recent years and is poised to transform many facets of healthcare, including medical education [2, 12, 26, 32, 34, 43, 46, 49, 52]. AI, which can be defined as ‘intelligence demonstrated by machines’ (Fig. 1) [1], has primarily been pioneered in ‘data-driven’ specialties like radiology, oncology, neurology, and pathology [30, 48, 52]. While AI is already being utilised in orthopaedics for various purposes, notably preoperative risk stratification [24, 31, 36], outcome prediction [27], diagnostic image analysis, preoperative planning [42, 45, 55], optimisation of postoperative rehabilitation [10, 17], and automation of administrative tasks [25, 57], integration of AI into this field is still in its infancy [11, 30]. Thus, the sentiment of the next generation of practitioners will be pivotal in defining the way this novel technology will shape the orthopaedic profession.

A number of studies have evaluated the attitude of medical students [4, 5, 7, 19, 33, 40, 53] and non-orthopaedic physicians [15, 39] towards AI [3, 4, 14, 37, 56], mostly focusing on radiology [5, 38, 40, 41, 44, 56]. Existing literature reports favourable views towards AI, as well as insufficient expertise [3, 9, 14, 39, 56] paired with inadequate AI education in medical training [13, 14, 21, 54], generating a strong demand for increased integration of AI into medical curricula [7, 8, 21, 44, 47, 54, 56].

In contrast, there is a notable deficiency in research focusing on orthopaedic surgery [14], particularly concerning key issues such as fear of replacement [4, 5, 7, 19, 20, 37, 40, 51], the perception of AI as an identity threat [22], and the impact of recent developments in AI technology on the career choices of medical students in favour of or against orthopaedic surgery [4, 16, 38, 41, 44].

The purpose of this study was to evaluate the perspectives and proficiency of prospective orthopaedic surgeons regarding AI, analysing how gender, AI knowledge, and technical inclination influence views on AI. Additionally, the extent to which recent AI advancements swayed career decisions towards or away from the orthopaedic field was assessed. The initial hypothesis posited that participants, despite possessing relatively limited expertise, would express a favourable sentiment towards AI.

## Material and methods

This anonymous, multinational, cross-sectional survey study was designed and conducted in accordance with the Checklist for Reporting Results of Internet E-Surveys (CHERRIES) [18]. The survey was created and conducted using SurveyMonkey (SurveyMonkey Inc., San Mateo, CA, USA), and disseminated among student members of the largest orthopaedic associations in Germany, Switzerland, and Austria (i.e., the AGA Society for Arthroscopy and Joint Surgery, the DGOU German Society for Orthopaedics and Trauma Surgery, and the DVSE D-A-CH Association for Shoulder and Elbow Surgery). Students within the AGA Association for Arthroscopy and Joint Surgery and student members of the DGOU were contacted via email. Furthermore, the survey was disseminated via the social media channels of the AGA Society, and the DVSE. Proficiency in the German language was a prerequisite for participation and the intent to pursue a career in orthopaedics was defined as an inclusion criterion.

The survey underwent comprehensive testing to ensure accuracy, usability, and technical functionality. As such, after an initial version was implemented, the preliminary questionnaire was circulated among all co-authors and independently reviewed. After a thorough discussion, any necessary adjustments were made, and the final version of the questionnaire was implemented. The survey provider ensured that each participant could only complete the questionnaire once, based on respondents’ IP addresses. In addition to the publication of the survey on social media platforms as mentioned above, the initial outreach to AGA Society student members occurred via email, followed by reminders on the 7th, 16th, 23rd, and 37th day after initial contact. DGOU student members were contacted once via mail. The survey, active for 40 days, welcomed voluntary responses without password protection or incentives. Participants were briefed on the survey’s objective, estimated duration, and the principal investigator’s identity. Institutional review board approval was not required given the study’s nature [28].

### Survey design

The survey comprised 18 questions, designed in accordance with previously published questionnaires [15, 33, 37, 39, 40, 51]. Questions were presented in a fixed order on a single page, without adaptive questioning. Both single choice and multiple-choice questions were employed. Participants were able to skip questions. All questions included a ‘no response’ answer option, except for question 12. Respondents were able to review and modify their responses prior to submission. The questionnaire was designed and distributed in German. An English translation of the survey questionnaire is provided in Supplementary Table 1.

Questions 1 and 2 sought general demographic data, capturing gender and year in medical school, respectively. Questions 3 and 5 were designed to assess commitment to pursuing a career in orthopaedics, as well as prior exposure to the orthopaedic specialty. Questions 4 and 6 captured self-reported technical affinity and AI literacy. Question 12 was a multiple-choice question with three correct and two incorrect answer options, intended to objectively assess basic knowledge about AI. Question 7 assessed opinions on the impact of AI in orthopaedics and anticipated timeframe thereof. Question 8 captured fear of replacement across (i) core medical tasks, e.g., performing surgery, (ii) other medical tasks, e.g., radiological assessment, anamnesis, and (iii) administrative tasks, e.g., documentation. Question 9 assessed interest in using specific AI applications. Question 10 was designed to evaluate concerns about AI (comprehensive list of likely areas of concern based on existing literature [9, 14, 33, 56]), while question 11 assessed the perception of AI as an ‘identity threat’ [22], as well as enthusiasm towards AI. Questions 13 and 14 were intended to assess opinions about the status quo of AI in medical training, as well as demand for specific educational opportunities (comprehensive list of possible settings, in accordance with previously published data [4, 20, 29]). Question 15 assessed the impact of AI on the choice of pursuing a residency in orthopaedics. Questions 16–18 were designed to evaluate the influence of recent advancements in AI technology on studying techniques, perceived devaluation of memorising detailed medical facts, and current use of AI in medical training.

### Subgroup analyses

Given the cohort size, the number of possible comparisons in the subgroup analyses was limited due to the risk of a type 1 error. Thus, only the following comparisons were performed and included in the final analysis: (i) correlation between measured AI literacy and years in medical school, (ii) influence of self-reported technical affinity on sentiments towards AI, (iii) influence of gender on sentiments towards AI, and (iv) influence of measured AI literacy on sentiments towards AI. Answers of ‘no response’ were treated as missing values and excluded from the corresponding analyses.

### Statistical analysis

Statistical analysis was performed using SPSS version 26.0 (IBM SPSS, New York, USA). Respondents indicating no intent to pursue a career in orthopaedics and trauma surgery were excluded from the analysis. Incomplete datasets were excluded from the corresponding analyses. Categorical variables are reported as counts and percentages. Continuous variables are reported as means and standard deviations or medians and interquartile ranges in case of normal/non-normal distributions, respectively. Data were assessed for normality using histograms, Q–Q plots, Kolmogorov–Smirnov/Shapiro–Wilk tests, and equal variance (Levene’s test). Subgroups were compared using the Mann–Whitney U test or Spearman’s rank correlation coefficient, as statistically appropriate. The significance threshold was set at p < 0.05. No alpha adjustment was performed. For question 12, a correctness score was calculated, adding one point for each marked correct answer option, and subtracting one point for each marked incorrect answer option, to assess both pre-existing AI knowledge, as well as misconceptions about AI. Unmarked correct answer options were not penalised. An a priori power analysis was not performed, as this study was constrained by the number of eligible participants.

## Results

**Fig. 2:**
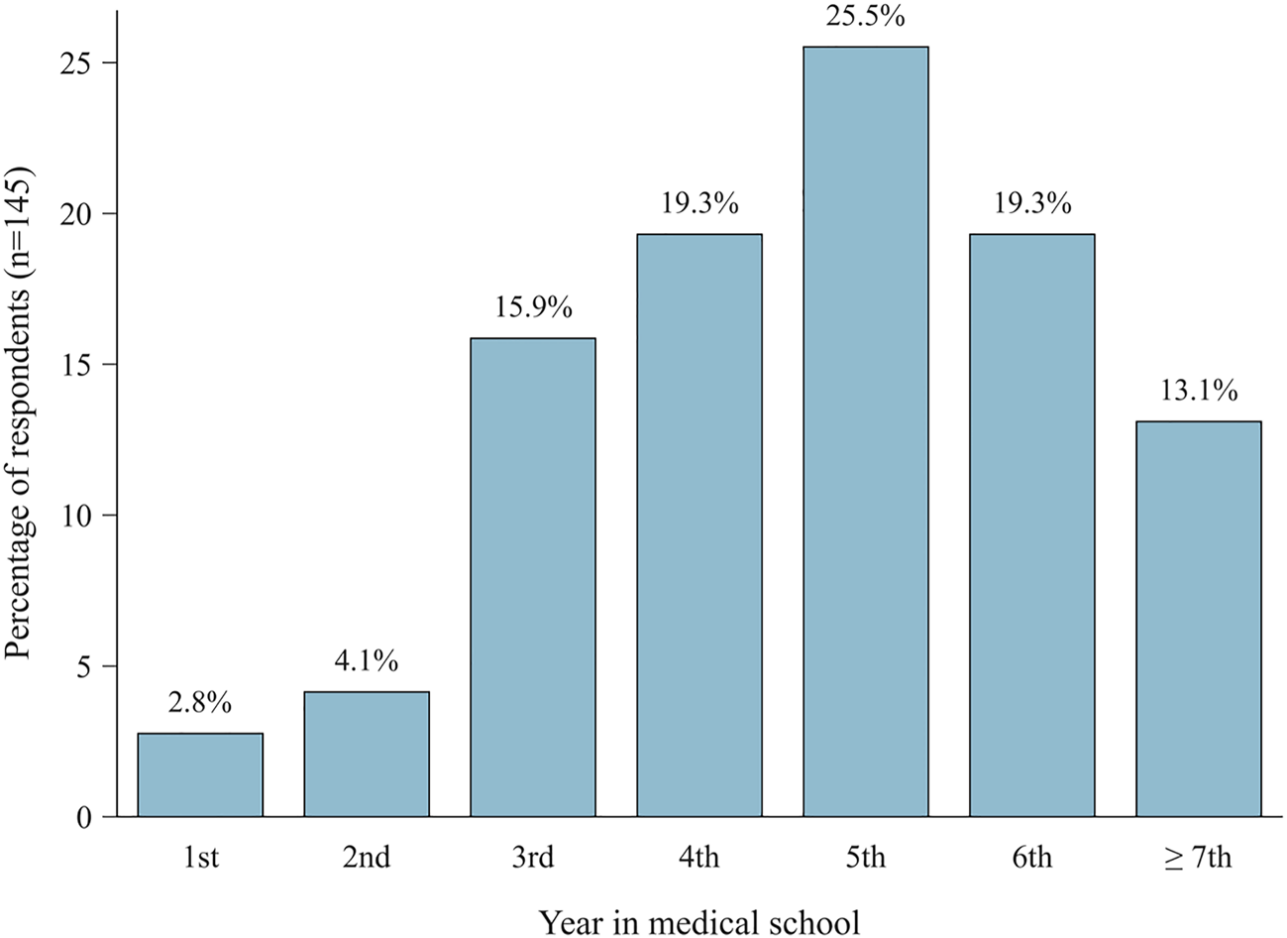
Respondents’ stage of medical education. Bar chart displaying respondents’ stage in medical education (n = 145)

**Fig. 3:**
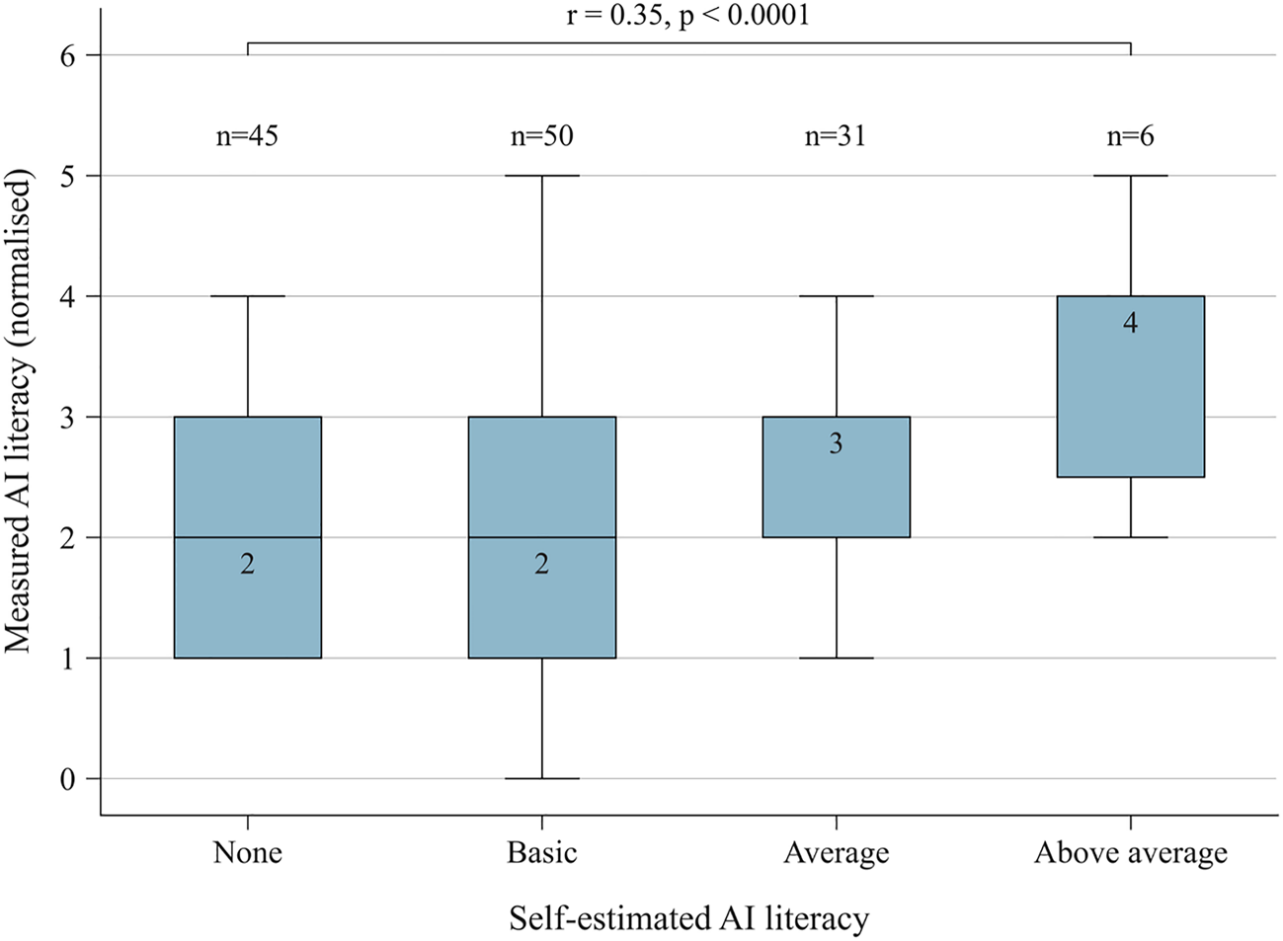
Relationship between subjective and objective AI literacy. Box plot illustrating the relationship between subjective and objective AI literacy. Variables were compared using Spearman’s ranked correlation coefficient (r = 0.35, p < 0.0001), indicating a significant positive correlation. Data labels indicate medians.* AI* artificial intelligence

A total of 174 medical students participated in this survey, constituting an approximate response rate of 19%. Respondents who reported no intent to pursue a career in orthopaedics were excluded from the analysis. Thus, the final cohort comprised exclusively aspiring orthopaedic surgeons (n = 150, 48% female, 52% male). The distribution of respondents’ years in medical school is detailed in Fig. 2. Most participants reported extensive prior exposure to orthopaedics (Table 1). Respondents predominantly reported low technical affinity (Table 1), as well as limited knowledge about AI in medicine (cf. Fig 3, Supplementary Table 2). The mean correctness score for measured AI literacy (as per question 12), normalised to 0–1, was 0.43 ± 0.22 (n = 133, cf. Table 2).

**Table 1:** Technical aptitude and prior exposure to orthopaedics

Question 4—“How would you rate your technical skills in areas such as IT/computer science (e.g., programming, data analysis, network/system administration, software development)?”		
Answer option	n	%
No/basic interest and no knowledge	19	12.7
Basic interest, but limited knowledge	86	57.3
Interest and self-taught knowledge, but no formal education	17	11.3
Occasional engagement in spare time, but no formal education	19	12.7
In-depth engagement and practical experience, but no formal education	7	4.7
Degree in or currently enrolled in a technical field (e.g., computer science, bioinformatics, data science)	2	1.3
No answer	0	0

Question 5—“What exposure have you had to the field of orthopaedics and trauma surgery? (multiple choice)”		
Answer option	n	%
Member in a professional society	112	74.7
Regular assistance in the OR	107	71.3
Internship	130	86.7
Intern year	44	29.3
Publication in orthopaedics/trauma surgery	24	16
Elective in medical school	67	44.7
Dissertation in orthopaedics/trauma surgery	74	49.3
No prior exposure	0	0
No answer	2	1.3

Raw counts and percentages of responses to questions 4 and 5, assessing self-reported technical aptitude and prior exposure to the field of orthopaedics

*IT* Information technology; *OR* operating room

**Table 2:** Measured and self-reported AI literacy

Question 12—“Which of the following statements are correct?”			
Answer option	Correct	n	%
Deep learning is a sub-area of machine learning; machine learning is a sub-area of artificial intelligence	Yes	69	51.9
AI algorithms learn from data and make decisions based on pre-programmed rules	No	105	78.9
Supervised learning means that an AI-algorithm is trained on annotated data	Yes	37	27.8
Overfitting means that an AI-model performs badly on the training data	No	8	6.02
Convolutional neural networks are a class of AI-models that are predominantly used for image analysis	Yes	29	21.8

Raw counts and percentages of responses to question 12, objectively assessing basic AI knowledge (n = 133). Percentages represent the proportion of respondents who selected the respective answer option, not the proportion of correct responses

*AI* Artificial intelligence

**Fig. 4:**
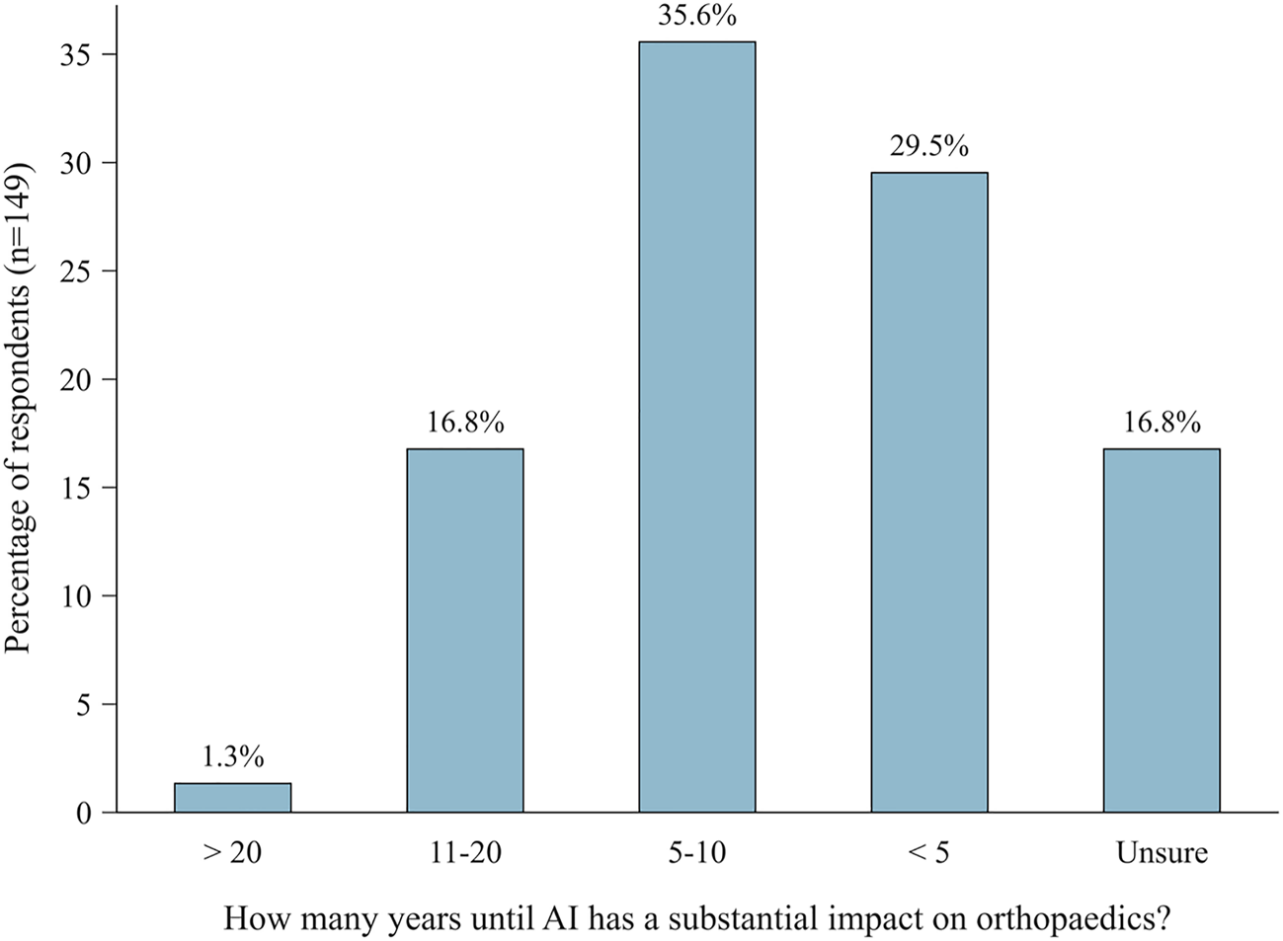
Anticipated impact of AI on orthopaedics. Bar chart displaying the anticipated timeframe until AI has a substantial impact on the practice of orthopaedics (n = 149). *AI* artificial intelligence

All respondents expected AI to have a noticeable impact on orthopaedics, with most projecting a timeframe of 5–10 years (35.3%) or < 5 years (29.3%) (Fig. 4). Most respondents thought AI would not replace human professionals, but rather ‘comprehensively assist’ them across (i) ‘core medical tasks’, e.g., performing surgery (89.3%), (ii) ‘other medical tasks’, e.g., radiological assessment or anamnesis (82%), and (iii) ‘administrative tasks’, such as documentation (62%). Replacement was considered most likely regarding administrative tasks (cf. Table 3). ‘AI-powered implant planning’, ‘AI-powered administration and automated documentation’, and ‘AI-powered diagnostic image analysis’ were identified as the most relevant clinical applications of AI (Table 4).

**Table 3:** Replacement versus assistance

Question 8—“How do you estimate the impact of AI in orthopaedics in the next 10 years in terms of clinical and administrative activities?”					
	Replacement of physicians/medical personnel	No replacement, but comprehensive assistance of physicians/medical personnel	Neither replacement, nor assistance of physicians/medical personnel	n.a	Total
Core medical tasks, e.g., performing surgery	0.7%	89.3%	8.7%	1.3%	100%
	1	134	13	2	150
Other medical tasks (radiological assessment, anamnesis, etc.)	15.3%	82%	1.3%	1.3%	100%
	23	123	2	2	150
Administrative tasks (documentation, drafting doctor’s notes, etc.)	34.7%	62%	2.7%	0.7%	100%
	52	93	4	1	150

Raw counts and percentages of responses to question 8, assessing views on the risk of replacement of human professionals by AI (n = 150). Participants were able to choose one answer option per row

*AI* Artificial intelligence; *n.a.* no answer

**Table 4:** Promising applications of AI in orthopaedics

Question 9—“If you were a practicing orthopaedic/trauma surgeon today, which specific AI tools would you be most likely to use? (multiple choice)”		
Answer option	n	%
AI-powered anamnesis and diagnostics software	69	46
AI-powered image analysis for diagnostics and classification (e.g., of fractures)	122	81.3
AI-powered implant planning	128	85.3
AI-powered treatment efficacy prediction	67	44.7
AI-powered robotics systems for precision surgery	79	52.7
AI-powered postoperative rehabilitation plans and progress analysis	86	57.3
AI-powered monitoring for early detection of complications	89	59.3
AI-powered precision medicine (e.g., patient specific implants, individualized surgery planning, personalized pain therapy)	76	50.7
AI-powered communication tools to improve patient-doctor-interactions	32	21.3
AI-powered administration and automated documentation	126	84
AI in orthopaedic and trauma surgery research (assistance for literature searches, scientific writing, etc.)	88	58.7
None	0	0
No answer	2	1.3
Something else	0	0

Raw counts and percentages of responses to question 9, assessing potential applications of AI in orthopaedics which respondents identified to be most promising (n = 150)

*AI* Artificial intelligence

**Fig. 5:**
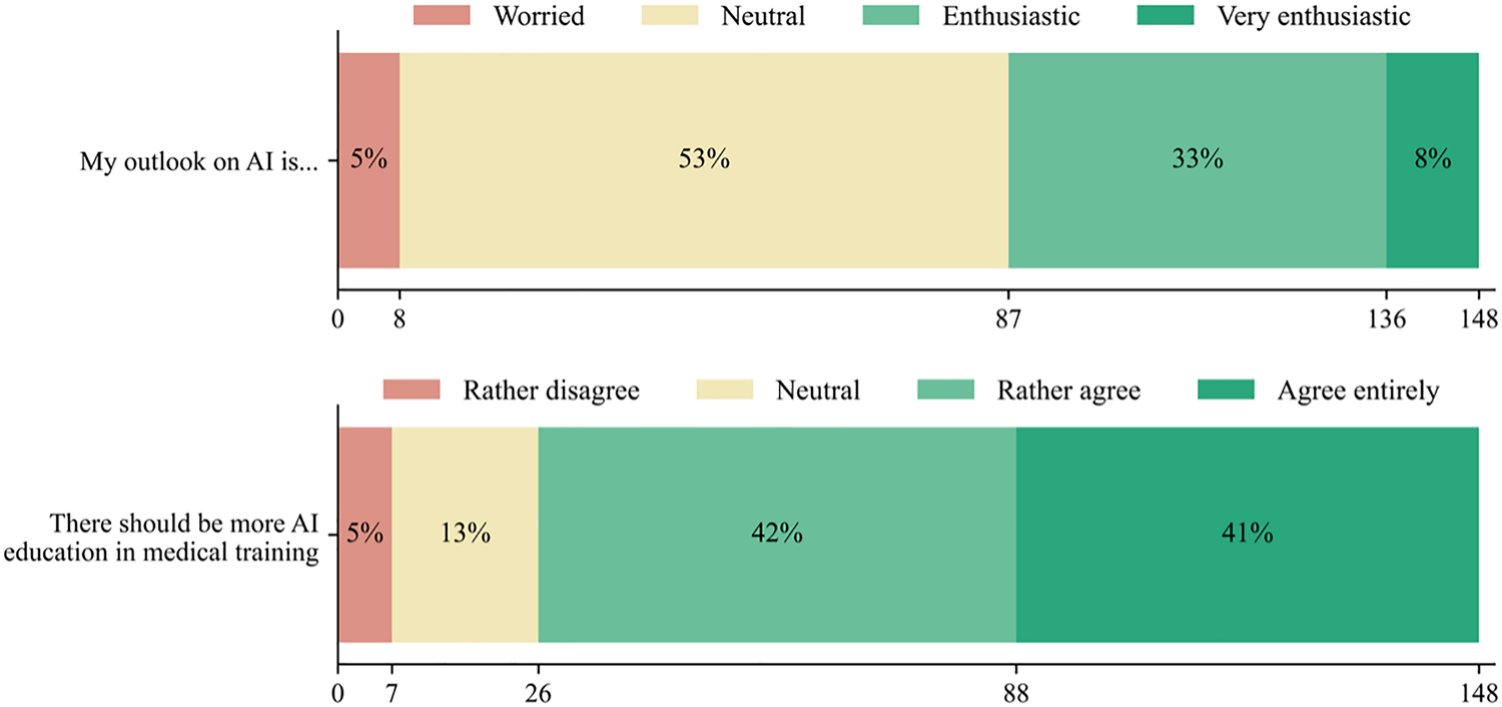
Positive sentiment towards AI and strong demand for AI education. Stacked bar charts illustrating attitudes towards AI (top), as well as demand for AI education in medical training (bottom). *AI* artificial intelligence

Concerns arose around (i) skill atrophy due to over-reliance on technology, (ii) legal aspects, and (iii) loss of human contact and empathy (Table 5). AI was not perceived as a significant identity threat, with most respondents reporting a ‘neutral’ or ‘enthusiastic’ stance towards AI (Fig. 5, top).

**Table 5:** Concerns about AI in orthopaedics

Question 10—“What concerns do you have regarding the integration of AI in orthopaedics/trauma surgery? (multiple choice)”		
Answer option	n	%
Ethical aspects	61	40.7
Legal aspects (e.g., liability)	102	68
Privacy and data security	63	42
Loss of human contact and empathy	84	56
Over-reliance on technology and skill atrophy	104	69.3
Lack of acceptance and trust by patients and medical professionals	54	36
Lack of integration of domain experts (orthopaedic/trauma surgeons) into the development process	60	40
Loss of autonomy	35	23.3
Potential amplification of biases	53	35.3
AI could replace orthopaedic/trauma surgeons	11	7.3
No concerns	3	2
No answer	1	0.7

Raw counts and percentages of responses to question 10, assessing concerns about specific aspects of the integration of AI into orthopaedics (n = 150)

*AI* Artificial intelligence

**Fig. 6:**
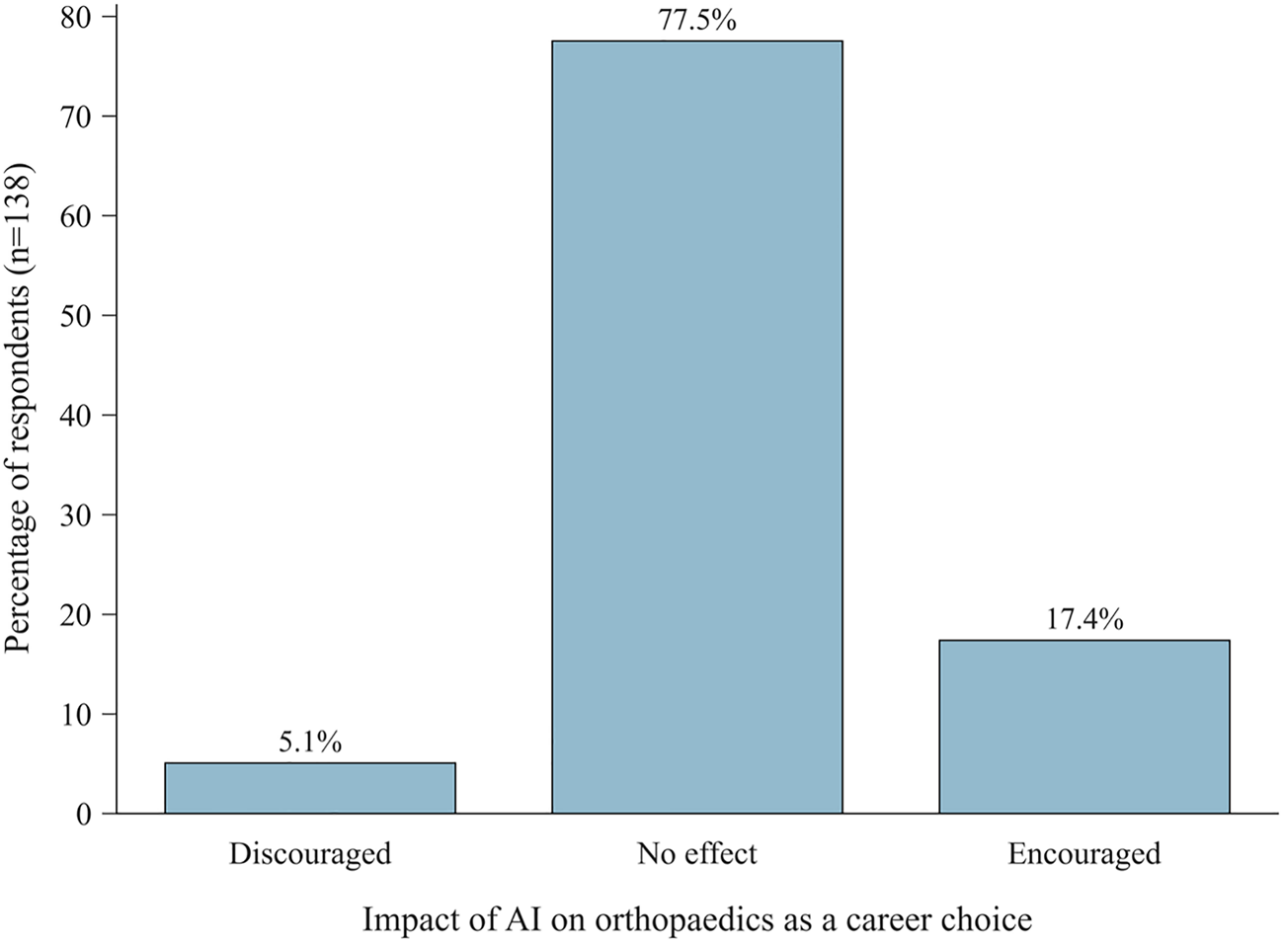
Impact of recent developments in the field of AI on orthopaedics as a career choice. Bar chart displaying the impact of recent advancements in AI technology on the career choices of aspiring orthopaedic surgeons. *AI* artificial intelligence; *n.a.* no answer

Most respondents agreed that AI should be a stronger component in medical training (Fig. 5, bottom). Interest in specific educational modalities is detailed in Table 6. The majority (72.8%) reported no influence of recent advancements in the field of AI on their career choices in favour of or against orthopaedic surgery. Of those reporting an influence, most felt encouraged to pursue a career in orthopaedics (Fig. 6). Most respondents (66.5%) indicated that current developments in AI, such as Large Language Models like ChatGPT passing medical exams with high grades, did not have an influence on their study techniques. Furthermore, the majority (65.8%) agreed that recent developments in AI technology did not devalue the importance of memorising detailed medical facts. Finally, most participants (57.1%) did not presently use AI tools such as ChatGPT for their own medical education.

**Table 6:** Demand for AI educational modalities

Question 14—“Which of the following educational modalities regarding AI would you be interested in? (multiple choice)”		
Answer option	n	%
Presentations about AI in orthopaedics/trauma surgery	114	76
Workshops and hands-on trainings about AI in orthopaedics/trauma surgery	115	76.7
Webinars and online panel discussions about AI in orthopaedics/trauma surgery	71	47.3
Medical school elective ‘AI in orthopaedics/trauma surgery’	78	52
Compulsory ‘medical AI’ course in medical school	48	32
Self-directed learning modules as an introduction to AI in orthopaedics/trauma surgery	45	30
Interdisciplinary seminars, connecting AI in orthopaedics/trauma surgery with other medical specialties	65	43.3
Cross-disciplinary conferences to foster the exchange of ideas and experiences regarding AI in orthopaedics/trauma surgery	61	40.7
Mentorship programs with experts in AI in orthopaedics/trauma surgery for individual education	50	33.3
Project-based learning, where students and experts work on real-world AI-related projects in orthopaedics/trauma surgery	60	40
Specialised AI certification programs for orthopaedic/trauma surgeons, to improve knowledge and skills	48	32
No answer	3	2
Something else	0	0

Raw counts and percentages of responses to question 14, assessing which specific educational modalities respondents were most interested in (n = 150)

*AI* Artificial intelligence

### Subgroup analyses

**Fig. 7:**
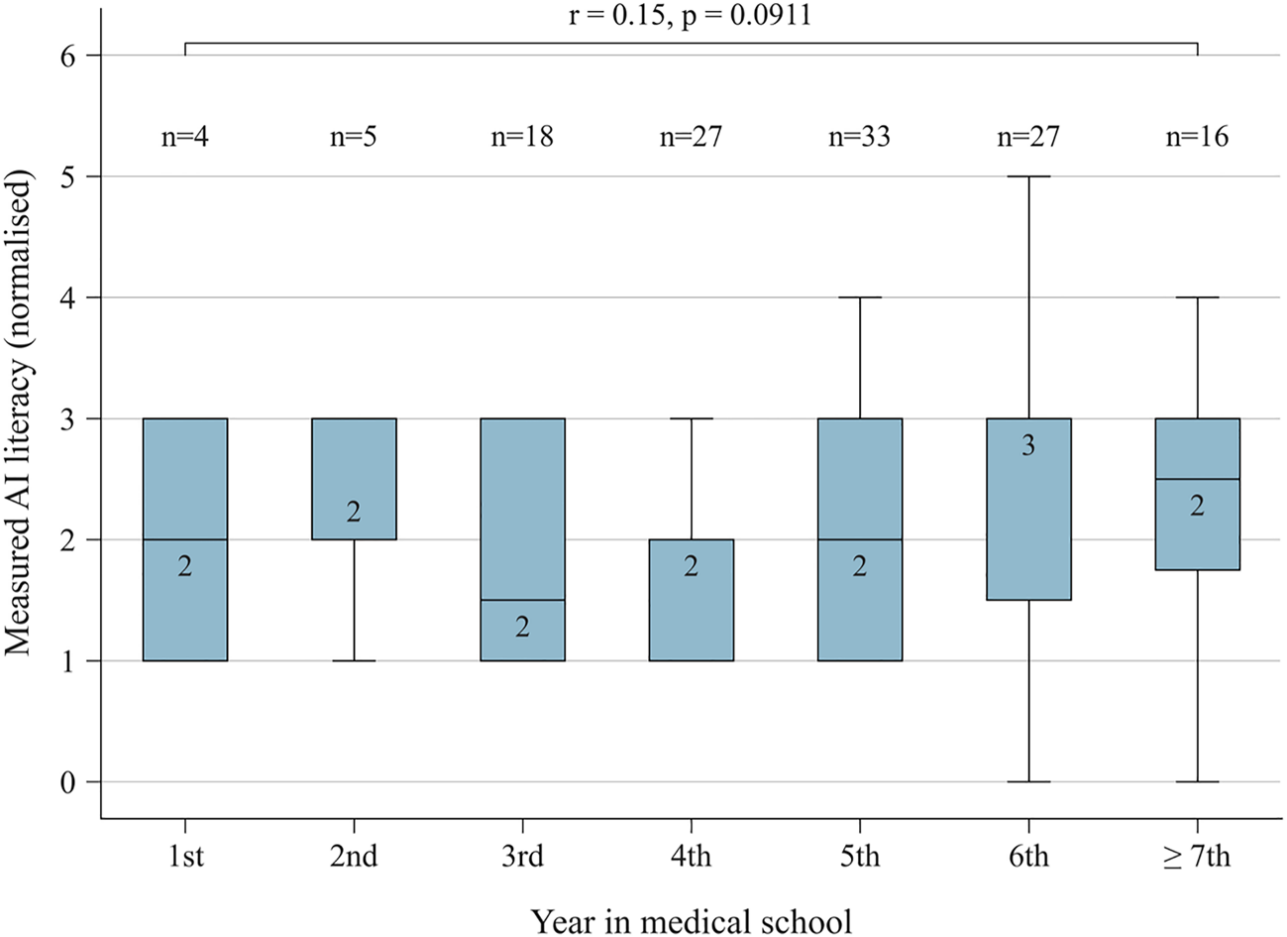
Relationship between year in medical school and AI literacy. Box plot illustrating the relationship between year in medical school and AI literacy. Variables were compared using Spearman’s ranked correlation coefficient (r = 0.15, p = 0.0911), indicating no significant correlation. Data labels indicate median values. *AI* artificial intelligence

Measured AI literacy (Spearman’s r = 0.21, p = 0.015) and self-reported technical affinity (Spearman’s r = 0.24, p = 0.003) correlated significantly with sentiment towards AI, while gender (p = 0.148) did not (Supplementary Tables 3, 4). Measured AI literacy did not increase throughout medical school (Spearman’s r = 0.15, p = 0.091, Fig. 7).

## Discussion

This study presents the following key findings: (i) aspiring orthopaedic surgeons display a positive sentiment towards AI, anticipating its significant impact on the orthopaedic profession in the near future, (ii) aspiring orthopaedic surgeons exhibit relatively low self-estimated and measured AI literacy, with no significant increase throughout medical school, but a strong demand for more AI-related educational offerings in medical training, and (iii) the career choices of aspiring orthopaedic surgeons in favour of or against the orthopaedic specialty are not significantly impacted by recent advancements in AI technology.

To date, rapid advancements in the field of AI have predominantly affected clinical specialties such as radiology, pathology, or cardiology [6]. However, there is a growing interest in AI among orthopaedic surgeons. As medical students are poised to be particularly impacted by the advent of AI in healthcare [33], the sentiment and expertise of these future doctors will be pivotal in shaping the field of orthopaedics, where AI integration is still nascent. Existing research has predominantly focused on practicing physicians [14, 15, 39] and while some studies have extended the scope of their investigation to medical students, those studies have mostly been focused on radiology [5, 14, 20, 38, 40, 41, 44] or have been specialty-agnostic [7, 19, 29, 33, 53]. To date, no studies have investigated the perspectives of aspiring orthopaedic surgeons on this important topic.

The findings of this survey study demonstrate that aspiring orthopaedic surgeons have a strong positive outlook on the forthcoming integration of AI into their specialty, aligning with the current literature, which reports generally positive attitudes [3–5, 7, 14, 21, 37, 41, 56]. In a recent multinational survey across 63 countries, 69.9% of respondents agreed that ‘AI developments will make medicine […] more exciting’ and 99% of respondents were ‘eager to incorporate AI in their future practice’ [7].

The present study revealed a comparatively low self-estimated AI literacy among aspiring orthopaedic surgeons, with most participants indicating either ‘no knowledge’ (34.9%) or ‘basic knowledge’ (39.6%), and correspondingly limited measured AI literacy, with an average normalised correctness score of 43%. This aligns with previous research, with many studies reporting a lack of expertise among medical students, physicians, and other healthcare professionals [3, 7, 9, 14, 56]. As such, McLennan et al. [33] report 64.4% of medical students are not feeling well informed about AI in medicine. Notably, we observed no significant increase in measured AI literacy throughout medical school, indicating a lack of effective transfer of basic AI knowledge in current medical school curricula. Correspondingly, a clear demand for AI in medical training in general, as well as the field of orthopaedic surgery in particular, was observed, with 81.9% of respondents indicating they would welcome the inclusion of more AI-related content in their medical education. Numerous studies highlight a lack of AI education in existing medical curricula [21, 44, 54] and underline the necessity to introduce AI-related content into medical training [4, 14, 16, 20, 23, 33, 53], with a significant demand among medical students [7, 8, 47, 54, 56]. Interestingly, measured AI literacy correlated significantly with sentiment towards AI, with more literate respondents being more likely to indicate enthusiasm about AI. This finding aligns with previous results, which revealed that high confidence in understanding of AI led to a decrease in anxiety levels [20].

Regarding specific modalities for optimal information transfer, respondents in this cohort were most interested in ‘workshops and hands-on training about AI in orthopaedics’ and ‘presentations on the topic of AI in orthopaedics’, while being least interested in ‘self-directed learning modules as an introduction to AI in orthopaedics’, suggesting a preference for expert-led modalities and practical ‘hands-on’ training. Comparably, in a study by Gong et al. [20], students were most interested in ‘expert opinions on the impact of AI’ (70.6%) and discussing ‘AI in pre-clinical radiology lectures’ (66.45%). However, only 29.75% were interested in ‘courses on AI’.

Several studies have postulated that AI might deter medical students from ranking certain specialties, most notably radiology [14, 20, 38, 41, 44]. A recent systematic review reported ‘almost half of all medical students feeling less enthusiastic’ about becoming radiologists because of AI [14]. Recent advancements in AI technology had minimal impact on the career choices of aspiring orthopaedic surgeons, indicating a lesser influence compared to counterparts in radiology and a potential higher inclination toward choosing the orthopaedic specialty in the future.

This cohort demonstrated a pronounced interest in AI applications related to ‘implant planning’, ‘administration and automated documentation’, and ‘diagnostic image analysis’, suggesting a prevailing inclination toward radiological applications, in line with existing evidence [35]. Advances in medical natural language processing, especially medical ‘foundation models’ (e.g., Google’s ‘Med-PaLM’ [43]) and even ‘generalist biomedical AI’ [34, 50], are poised to enable additional possibilities in the near future.

While AI is poised to revolutionise many aspects of healthcare, its integration into clinical practice is met with significant concerns. As such, fear of replacement by AI has been much discussed in recent literature [4, 5, 14, 19, 20, 37, 40, 51, 56]. The present study found that aspiring orthopaedic surgeons perceived AI predominantly as an assistive technology, with no significant fear of replacement, a perception mirrored in recent literature [5, 56]. The ‘manual’ nature of orthopaedics potentially contributes to these findings. Other notable concerns included issues such as over-reliance on technology, potential skill atrophy, legal considerations including liability, and a loss of human contact and empathy. These findings align with broader research identifying challenges such as lack of trust, operator dependence, increased duration of operative procedures, poor performance in unexpected situations or new data distributions, lack of empathy, ambiguous medico-legal responsibility, data security, privacy disclosure, and the need for legal regulation and oversight [9, 14, 33, 56]. Finally, digitalisation plays a significant role in this process. While it is not synonymous with AI, digitalisation and AI are closely intertwined, and sufficient digitalisation is a prerequisite für the adoption of AI in clinical practice.

Summarily, integrating AI into medical education will be crucial to ensure that future orthopaedic surgeons are sufficiently prepared for the advent of AI in healthcare. Ideally, curricula should cover AI fundamentals, with a focus on specific medical applications, to foster a basic understanding of fundamental AI techniques. Practical workshops, hands-on trainings, and expert-led seminars will be pivotal in this regard. Close collaboration with AI specialists will enhance learning and innovation. Additionally, teaching AI ethics, data privacy, and legal implications will ensure responsible use in clinical practice.

### Limitations

This study has several important limitations. First, the sample of this study only represents a fraction of the future generation of orthopaedic surgeons in the D-A-CH region and a possible non-response bias must be considered. Efforts were made to mitigate non-response bias, by sending regular email reminders, cooperating with multiple orthopaedic societies across three countries, and advertising the survey on social media. Second, response bias is a potential concern in this study. Efforts were made to address this issue through the inclusion of a ‘no response’ answer option, permitting the skipping of question, and providing participants the opportunity to review and modify responses prior to submission. Third, the external validity of these results may be limited, given the specificity of the cohort. Generalisation to healthcare systems beyond the D-A-CH region and other medical specialties may be limited. To validate these findings globally, external validation in international surveys is imperative. Fourth, the dynamic nature of the field of AI technology underscores that these results can only offer a snapshot of the current status quo, potentially requiring reassessment in the future. Finally, participants’ precise understanding/definition of the term AI, specifically in contrast to the concept of digitalisation, was not assessed, as this was not a primary goal of this study. However, discrepancies in the interpretation of this terminology seem to be common and may have affected these results.

## Conclusions

Future orthopaedic surgeons exhibit a favourable outlook on AI, foreseeing its significant influence in the near future. AI literacy remains relatively low and showed no improvement during medical school. There is notable demand for improved AI-related education. The choice of orthopaedics as a specialty appears to be robust against the sway of recent AI advancements.

## Supplementary Information

Below is the link to the electronic supplementary material.

Supplementary file1 (DOCX 23 KB)

Supplementary file2 (DOCX 15 KB)

Supplementary file3 (DOCX 17 KB)

Supplementary file4 (DOCX 19 KB)

## Data Availability

All data is available from the corresponding author upon reasonable request.

## Abbreviations

AI: Artificial intelligence
ML: Machine learning
GPT: Generative pre-trained transformer
LLM: Large language model
IT: Information technology
CHERRIES: Checklist for Reporting Results of Internet E-Surveys
CI: Confidence interval
