# Supplementary material for: Attitude of aspiring orthopaedic surgeons towards artificial intelligence: a multinational cross-sectional survey study
Source: Arch Orthop Trauma Surg. 2024 Aug 10;144(8):3541–52. doi: 10.1007/s00402-024-05408-0 (PMC11417067; doi:10.1007/s00402-024-05408-0)
Supplement: Supplementary file 1 — Supplementary file1 (DOCX 23 KB) [file 402_2024_5408_MOESM1_ESM.docx]

| ***Survey Questionnaire – AI in Orthopaedics*** |
| --- |
| **Question 1: Gender**   1. Female 2. Male 3. No response |
| **Question 2: Semester in medical school**   1. 1^st^ semester 2. 2^nd^ semester 3. […] 4. 13^th^ semester 5. > 13^th^ semester 6. No response |
| **Question 3: Are you planning on pursuing a career in orthopaedics?**   1. I am pursuing an operative career in this specialty. 2. I am pursuing a non-operative career in this specialty. 3. Yes, but I am unsure whether operative or non-operative. 4. No, I am pursuing a career in a different specialty. 5. I am not sure yet. 6. No response |
| **Question 4:** **How would you rate your technical skills in areas such as IT / computer science (e.g., programming, data analysis, network / system administration, software development)?**   1. No / limited interest and no knowledge. 2. Basic interest, but limited knowledge. 3. Interest and self-taught knowledge (e.g., online courses on *Coursera*), but no formal education. 4. Occasional engagement in spare time, but no formal education. 5. In-depth engagement and practical experience, but no formal education. 6. Degree or currently enrolled in a technical field, e.g., computer science, bioinformatics, data science. 7. No response |
| **Question 5: What exposure have you already had to the field of orthopaedics? (Multiple answers possible)**   1. Membership in professional society 2. Regular assistance in the OR 3. Internship / clinical rotation 4. Intern year 5. Publication in orthopaedics 6. Elective in medical school 7. Dissertation in orthopaedics 8. No exposure 9. No response |
| **Question 6: How would you describe your current knowledge about AI in medicine?**   1. No knowledge 2. Basic knowledge 3. Average knowledge 4. Above-average knowledge 5. Expert knowledge 6. No response |
| **Question 7: Do you think AI will have a noticeable impact on orthopaedics? If so, in what time frame?**   1. AI will have no noticeable impact. 2. AI will have a noticeable impact within > 20 years. 3. AI will have a noticeable impact within 11-20 years. 4. AI will have a noticeable impact within 5-10 years. 5. AI will have a noticeable impact within < 5 years. 6. AI will have a noticeable impact, but I am unsure how long it will take. 7. No response |
| **Question 8: How do you estimate the impact of AI in orthopaedics in the next 10 years in terms of clinical and administrative activities?**   1. **Core medical tasks (e.g., performing surgery)**    1. Replacement of physicians / medical personnel    2. No replacement, but comprehensive assistance of physicians / medical personnel    3. Neither replacement, nor assistance of physicians / medical personnel    4. No response 2. **Other medical tasks (e.g., radiological assessment, anamnesis, etc.)**    1. Replacement of physicians / medical personnel    2. No replacement, but comprehensive assistance of physicians / medical personnel    3. Neither replacement, nor assistance of physicians / medical personnel    4. No response 3. **Administrative tasks (documentation, drafting doctor’s notes, etc.)**    1. Replacement of physicians / medical personnel    2. No replacement, but comprehensive assistance of physicians / medical personnel    3. Neither replacement, nor assistance of physicians / medical personnel    4. No response |
| **Question 9: If you were a practicing orthopaedic surgeon today, which specific AI tools would you be most likely to use? (Multiple answers possible)**   1. AI-powered anamnesis and diagnostics software 2. AI-powered image analysis for diagnostics and classification (e.g., of fractures) 3. AI-powered planning of implant positioning and size 4. Predicting treatment outcomes and probability of success using AI 5. AI-powered robotics for precision surgery 6. AI-powered postoperative rehabilitation and progress analysis 7. AI-based monitoring for early detection of complications 8. AI-assisted precision medicine (e.g., personalised implants, individualised surgical planning, personalised pain management) 9. AI-powered communication tools to improve doctor-patient interactions 10. AI-powered administration and automated documentation 11. AI in orthopaedics / trauma surgery research (assistance with literature search, scientific writing, etc.) 12. None 13. No response 14. Something else (please specify): ___________________________ |
| **Question 10: What concerns do you have about the use of AI in orthopaedics? (Multiple answers possible)**   1. Ethical aspects 2. Legal aspects (e.g., liability) 3. Privacy and data protection 4. Loss of human contact and empathy 5. Dependence on technology and possible skill atrophy 6. Lack of acceptance / trust from patients and medical professionals 7. Lack of integration of domain experts (orthopaedic / trauma surgeons) into the development process 8. Loss of autonomy 9. Potential amplification of bias 10. AI could replace orthopaedic / trauma surgeons 11. None 12. No response |
| **Question 11: How do you feel about the integration of AI into orthopaedics in terms of your personal identity / role as a future physician?**   1. Very worried 2. Worried 3. Neutral 4. Enthusiastic 5. Very enthusiastic 6. No response |
| **Question 12: Which of the following statements are correct? (Multiple answers possible)**   1. **Deep learning is a sub-area of machine learning; machine learning is a sub-area of artificial intelligence.** 2. AI-algorithms learn from data and make decisions based on pre-programmed rules. 3. **Supervised learning means that an AI-algorithm is trained on annotated data.** 4. Overfitting means that an AI-model performs badly on the training data. 5. **Convolutional neural networks are a class of AI-models that are predominantly used for image analysis.** |
| **Question 13: I would like to see artificial intelligence covered and taught more in medical education (medical school, professional societies, etc.).**   1. Disagree entirely 2. Rather disagree 3. Neutral 4. Rather agree 5. Agree entirely 6. No response |
| **Question 14: Which of the following educational offerings on the topic of AI would you endorse / take advantage of? (Multiple answers possible)**   1. Presentations on AI in orthopaedics / trauma surgery 2. Workshops and hands-on training on AI in orthopaedics / trauma surgery 3. Webinars and online panel discussions on AI in orthopaedics / trauma surgery 4. Elective on AI in orthopaedics / trauma surgery in medical school curricula 5. Required module “Medical AI” in medical school curricula 6. Self-directed “Intro to AI” learning module 7. Interdisciplinary seminars, connecting AI in orthopaedics / trauma surgery with other specialties 8. Interdisciplinary conferences to exchange ideas and experiences regarding AI in orthopaedics / trauma surgery 9. Mentorship programs with experts on AI in orthopaedics / trauma surgery for individualised education 10. Project-based learning, where students and domain experts collaborate on a real-world AI project in orthopaedics / trauma surgery 11. Specialised AI certification for orthopaedic / trauma surgeons, to improve their skills and expertise 12. No response 13. Something else (please specify): ___________________________ |
| **Question 15: To what extent has the progressive integration of AI in medicine influenced your decision to pursue the specialty of orthopaedics?**   1. The progressive integration of AI encouraged me in my decision to pursue this specialty, because I believe that “manual” specialties will be more resistant to automation with AI. 2. The progressive integration of AI encouraged me in my decision to pursue this specialty, because I believe that AI will create interesting opportunities in this specialty. 3. The progressive integration of AI encouraged me in my decision to pursue this specialty for a different reason. 4. The progressive integration of AI discouraged me in my decision to pursue this specialty, because I fear AI might devalue my skills in this area. 5. The progressive integration of AI discouraged me in my decision to pursue this specialty, because I believe that AI can be leveraged better in other specialties. 6. The progressive integration of AI discouraged me in my decision to pursue this specialty, for a different reason. 7. The progressive integration of AI had no impact on my decision to pursue / not pursue this specialty. 8. No response |
| **Question 16: News that AI systems, such as ChatGPT, pass medical exams with good grades influence my learning strategies.**   1. Disagree entirely 2. Rather disagree 3. Neutral 4. Rather agree 5. Agree entirely 6. No response |
| **Question 17: Due to current developments in AI, it is becoming less and less important to memorise medical details.**   1. Disagree entirely 2. Rather disagree 3. Neutral 4. Rather agree 5. Agree entirely 6. No response |
| **Question 18: I already use AI tools (e.g., ChatGPT) for my own medical education.**   1. Disagree entirely 2. Rather disagree 3. Neutral 4. Rather agree 5. Agree entirely 6. No response |

**Supplementary Table 1: Survey Questionnaire.** English translation of the full questionnaire used in this survey study. The questionnaire was originally designed and distributed in German. For question 12, **bold formatting** is used to indicate correct answer options. *Abbreviations: OR, operating room; AI, artificial intelligence; IT, information technology.*
