# Supplementary material for: Attitude of aspiring orthopaedic surgeons towards artificial intelligence: a multinational cross-sectional survey study
Source: Arch Orthop Trauma Surg. 2024 Aug 10;144(8):3541–52. doi: 10.1007/s00402-024-05408-0 (PMC11417067; doi:10.1007/s00402-024-05408-0)
Supplement: Supplementary file 2 — Supplementary file2 (DOCX 15 KB) [file 402_2024_5408_MOESM2_ESM.docx]

| **Question 6 – “How would you describe your current knowledge about AI in medicine?”** | | |
| --- | --- | --- |
| **Answer Option** | **n** | **%** |
| No knowledge | 52 | 34.9 |
| Basic knowledge | 59 | 39.6 |
| Average knowledge | 32 | 21.5 |
| Above-average knowledge | 6 | 4 |
| Expert knowledge | 0 | 0 |
| No answer | 0 | 0 |

**Supplementary Table 2: Self-reported AI literacy.** Raw counts and percentages of responses to question 6, assessing self-reported AI literacy (n=149). *Abbreviations: AI, artificial intelligence.*
