# Supplementary material for: Attitude of aspiring orthopaedic surgeons towards artificial intelligence: a multinational cross-sectional survey study
Source: Arch Orthop Trauma Surg. 2024 Aug 10;144(8):3541–52. doi: 10.1007/s00402-024-05408-0 (PMC11417067; doi:10.1007/s00402-024-05408-0)
Supplement: Supplementary file 3 — Supplementary file3 (DOCX 17 KB) [file 402_2024_5408_MOESM3_ESM.docx]

|  |  | ***Question 11: How do you feel about the integration of AI into orthopaedics in terms of your personal identity / role as a future physician?*** | | | | | |
| --- | --- | --- | --- | --- | --- | --- | --- |
|  |  | Very worried | Worried | Neutral | Enthusiastic | Very Enthusiastic | *Total* |
| ***Question 4: How would you rate your technical skills in areas such as IT / computer science (e.g., programming, data analysis, network / system administration, software development)?*** | No / limited interest and no knowledge | 0.00%  (n=0) | 5.26% (n=1) | **68.4% (n=13)** | 26.3% (n=5) | 0.00% (n=0) | *100%*  *(n=19)* |
|  | Basic interest, but limited knowledge | 0.00%  (n=0) | 5.88% (n=5) | **57.6% (n=49)** | 30.6% (n=26) | 5.88% (n=5) | *100%*  *(n=85)* |
|  | Interest and self-taught knowledge, but no formal education | 0.00%  (n=0) | 12.5% (n=2) | **43.8% (n=7)** | 18.8% (n=3) | 25.0% (n=4) | *100%*  *(n=16)* |
|  | Occasional engagement in spare time, but no formal education | 0.00%  (n=0) | 0.00% (n=0) | 36.8% (n=7) | **63.2% (n=12)** | 0.00% (n=0) | *100%*  *(n=19)* |
|  | In-depth engagement and practical experience, but no formal education | 0.00%  (n=0) | 0.00% (n=0) | 28.6% (n=2) | **42.9% (n=3)** | 28.6% (n=2) | *100%*  *(n=7)* |
|  | Degree or currently enrolled in a technical field, e.g., computer science, bioinformatics, data science | 0.00%  (n=0) | 0.00%  (n=0) | **50.0% (n=1)** | 0.00% (n=0) | **50.0%**  **(n=1)** | *100%*  *(n=2)* |
|  | *Total* | *0* | *8* | ***79*** | *49* | *12* | *148* |

**Supplementary Table 3: Influence of self-reported technical aptitude on sentiment towards AI.** Cross-tabulation of self-reported technical aptitude and sentiment towards AI, as it pertains to personal identity / role as a future physician. Responses of ‘no answer’ were treated as missing values and removed from this analysis (omitted here). Ordinal values were compared using Spearman’s rank correlation coefficient (r=0.24, p=0.003). **Bold formatting** is used to indicate the most frequent answer option in each row. *Abbreviations: AI, artificial intelligence; IT, information technology.*
