# Supplementary material for: Attitude of aspiring orthopaedic surgeons towards artificial intelligence: a multinational cross-sectional survey study
Source: Arch Orthop Trauma Surg. 2024 Aug 10;144(8):3541–52. doi: 10.1007/s00402-024-05408-0 (PMC11417067; doi:10.1007/s00402-024-05408-0)
Supplement: Supplementary file 4 — Supplementary file4 (DOCX 19 KB) [file 402_2024_5408_MOESM4_ESM.docx]

|  |  | ***Question 11: “How do you feel about the integration of AI into orthopaedics / trauma surgery, in terms of your personal identity / role as a future physician?”*** | | | | | |
| --- | --- | --- | --- | --- | --- | --- | --- |
|  |  | Very worried | Worried | Neutral | Enthusiastic | Very Enthusiastic | *Total* |
| ***Question 12: Measured AI literacy (raw correctness score)*** | -2 | 0.00%  (n=0) | 0.00% (n=0) | 0.00% (n=0) | **100% (n=2)** | 0.00% (n=0) | *100%*  *(n=2)* |
|  | -1 | 0.00%  (n=0) | 6.82% (n=3) | **61.4% (n=27)** | 27.3% (n=12) | 4.55% (n=2) | *100%*  *(n=44)* |
|  | 0 | 0.00%  (n=0) | 2.94% (n=1) | **61.8% (n=21)** | 29.4% (n=10) | 5.88%  (n=2) | *100%*  *(n=34)* |
|  | 1 | 0.00%  (n=0) | 5.13% (n=2) | 41.0%  (n=16) | **46.2% (n=18)** | 7.69% (n=3) | *100%*  *(n=39)* |
|  | 2 | 0.00%  (n=0) | 10.0% (n=1) | **30.0% (n=3)** | **30.0% (n=3)** | **30.0% (n=3)** | *100%*  *(n=10)* |
|  | 3 | 0.00%  (n=0) | 0.00% (n=0) | 0.00% (n=0) | **66.7% (n=2)** | 33.3% (n=1) | *100%*  *(n=3)* |
|  | *Total* | *0* | *7* | ***67*** | *47* | *11* | *132* |

**Supplementary Table 4: Influence of measured AI literacy on sentiment towards AI.** Cross-tabulation of measured AI literacy (raw correctness score between -2 and 3, as assessed by question 12) and participants’ sentiment / enthusiasm towards AI, as it pertains to their personal identity / role as a future physician. Responses of ‘no answer’ were treated as missing values and removed from this analysis (omitted here). Ordinal values were compared using Spearman’s rank correlation coefficient (r=0.21, p=0.015). **Bold formatting** is used to indicate the most frequent answer option in each row. *Abbreviations: AI, artificial intelligence.*
